# Core Components and Implementation Determinants of Multilevel Service Delivery Frameworks Across Child Mental Health Service Settings

**DOI:** 10.1007/s10488-023-01320-8

**Published:** 2023-12-20

**Authors:** Stephanie A. Moore, Jennifer McGrory Cooper, JoAnne Malloy, Aaron R. Lyon

**Affiliations:** 1 School of Education, University of California Riverside, Riverside, CA 92521 USA; 2 Division of Psychological and Educational Services, Fordham University, New York, USA; 3 Institute on Disability, College of Health and Human Services, University of New Hampshire, Durham, USA; 4 Department of Psychiatry and Behavioral Sciences, University of Washington, Seattle, USA

**Keywords:** Child mental health, Core components, Implementation determinants, Service delivery frameworks

## Abstract

Multilevel service delivery frameworks are approaches to structuring and organizing a spectrum of evidence-based services and supports, focused on assessment, prevention, and intervention designed for the local context. Exemplar frameworks in child mental health include positive behavioral interventions and supports in education, collaborative care in primary care, and systems of care in community mental health settings. Yet, their high-quality implementation has lagged. This work proposes a conceptual foundation for multilevel service delivery frameworks spanning diverse mental health service settings that can inform development of strategic implementation supports. We draw upon the existing literature for three exemplar multilevel service delivery frameworks in different child mental health service settings to (1) identify core components common to each framework, and (2) to highlight prominent implementation determinants that interface with each core component. Six interrelated components of multilevel service delivery frameworks were identified, including, (1) a systems-level approach, (2) data-driven problem solving and decision-making, (3) multiple levels of service intensity using evidence-based practices, (4) cross-linking service sectors, (5) multiple providers working together, including in teams, and (6) built-in implementation strategies that facilitate delivery of the overall model. Implementation determinants that interface with core components were identified at each contextual level. The conceptual foundation provided in this paper has the potential to facilitate cross-sector knowledge sharing, promote generalization across service settings, and provide direction for researchers, system leaders, and implementation intermediaries/practitioners working to strategically support the high-quality implementation of these frameworks.

## Introduction

Despite rising rates of youth mental health disorder and deaths by suicide (Houtrow et al., 2014; Miron et al., 2019), few children and adolescents receive needed mental health treatment (Costello et al., 2014; Merikangas et al., 2011; Simon et al., 2015). Discrepancies in receipt of high-quality care are further pronounced for youth of color and families living in communities experiencing high rates of poverty (Alegría et al., 2015; Hodgkinson et al., 2017; Locke et al., 2017). When left untreated, mental health disorders that arise in childhood and adolescence persist and contribute to later deleterious outcomes (Office of the Surgeon General, 2021). Accordingly, increasing youths’ access to high-quality prevention, early intervention, and mental health treatment services is critical.

### Multilevel Service Delivery Frameworks

Schools, outpatient settings, and primary care are the most common loci of mental health care for youth who receive services (Duong et al., 2021). Across these principal youth mental health service sectors, service delivery frameworks designed to address the full range of youth mental health needs (i.e., via prevention, early intervention, and treatment) are championed as best practices for increasing access to care and improving outcomes—exemplars include positive behavioral interventions and supports (PBIS) in education settings (e.g., Bradshaw et al., 2009; Eiraldi et al., 2019), the collaborative care model (CoCM) in primary care settings (e.g., Goodrich et al., 2013; Richardson et al., 2017), and system of care (SoC) in community mental health settings (e.g., Stroul et al., 2008, 2021). As used here, these *multilevel service delivery frameworks* are defined as approaches to structuring and organizing a spectrum of research-based services and supports, focused on assessment, prevention, and intervention matched to the needs of the target population and appropriate for the service context. Although the application of these frameworks is common across many youth (and adult) service sectors, integration of their disparate literatures to identify common components or generalizable findings about their implementation is needed.

The features and services that comprise multilevel service delivery frameworks are situated at multiple tiers—including practices of varied intensity targeted toward whole populations, small groups, or individuals (i.e., tiered service delivery or primary, secondary, and tertiary prevention). Each tier may also focus on multiple actors within that service system (e.g., clients, providers, administrative units, and policymaking/legislative bodies). We use the term “frameworks” to be inclusive of the varied components within these approaches to multilevel service delivery, including discrete intervention and assessment methods as well as the structures that facilitate their delivery. Integral to multilevel service delivery frameworks is the specification of how components relate to one another and are administratively managed. For example, services of varying levels of intensity that occur across multiple entities may be coordinated by teams who use data to guide decision-making. Although multilevel service delivery frameworks are not limited to youth mental health services (e.g., CoCM has been implemented far more extensively with adults than with youth), we focus on child-serving treatment settings for myriad reasons including the current youth mental health crisis (Office of the Surgeon General, 2021) and because there are several well-articulated examples of multilevel service delivery frameworks for the primary settings in which youth typically access mental health care.

### Implementation of Multilevel Frameworks

Multilevel service delivery frameworks hold great potential for addressing a wide range of youth mental health needs, but this potential is unlikely to be achieved without widespread adoption and high-quality implementation within their respective service settings (Durlak & Dupree, 2008). Frameworks such as CoCM, PBIS, and SoC are increasingly adopted (AIMS Center, 2021; Center on PBIS, 2023a; Stroul et al., 2021), however, their implementation is fraught with challenges. For example, insufficient organizational capacity, piecemeal implementation of key features (i.e., intervention and assessment), and ineffective development and/or use of data systems (e.g., to inform intervention selection or monitor progress; e.g., Fabiano & Evans, 2019; Overbeck et al., 2016) can result in implementation outcomes that differ substantially from the intended model (e.g., Molloy et al., 2013). When considering multilevel service delivery frameworks, implementation outcomes such as “fidelity” might refer either to *intervention* fidelity—focused on the delivery of service-recipient-facing component practices (also called intervention integrity or treatment fidelity)—or *implementation strategy* fidelity—the extent to which implementation practices and techniques were delivered as intended. Given the complexity of these frameworks, both are important in understanding child outcomes (Saldana, 2014; Sanetti et al., 2021).

Similar to more circumscribed evidence-based interventions (Durlak & Dupree, 2008; Lawson et al., 2018), identifying core components of multilevel service delivery frameworks can lay the groundwork for tracking and evaluating implementation of these frameworks. For example, identifying which components service systems are most (or least) often implementing with fidelity (intervention or strategy) allows us to better understand how a component’s implementation impacts youth mental health and service outcomes. Whereas scholars have begun to explicate and investigate key features of individual multilevel service delivery frameworks, such as CoCM in primary care (Bao et al., 2016; Wood et al., 2017) and PBIS in schools (Molloy et al., 2013), this work is framework- and setting-specific, and thus limited in its generalizability to other frameworks and settings. Identifying multilevel service delivery frameworks as a common phenomenon across mental health service sectors is an important next step. Despite the pertinence of prior research for enhancing implementation of the targeted framework, research that spans service settings and frameworks may enable knowledge sharing across scholars and professionals within different service systems. In this paper, we argue that synthesizing the evidence relative to core features of varied multilevel service delivery frameworks has the potential to inform a larger understanding of those frameworks and, in doing so, improve their implementation and sustainment across settings.

As implementation of service delivery frameworks has been found to vary by core component (e.g., Molloy et al., 2013), understanding how their core features interface with implementation barriers and facilitators (i.e., determinants) is also critical for supporting their high-quality implementation. Implementation strategies are likely to be most effective when strategies are tailored to the implementation determinants that are most salient for the target innovation and its context (Baker et al., 2015; Lau et al., 2015; Powell et al., 2017). Research in support of this goal is emerging for exemplar multilevel service delivery frameworks. For example, a systematic review of CoCM for depression conducted by Wood et al. (2017) identified barriers and facilitators to the implementation of specific components of CoCM (e.g., barriers to multi-professional teamwork included organization culture and negative staff attitudes to change, whereas facilitators included staff champions and peer learning and support). Scholars have also identified and investigated core components (e.g., Molloy et al., 2013; Center on PBIS, 2015) and salient implementation determinants (e.g., Fox et al., 2022) for PBIS. However, to our knowledge, research has yet to link implementation determinants to core features of exemplar frameworks in school or community care settings nor to the core components shared by multilevel service delivery frameworks. Given the research in primary care settings that indicates important determinants vary by component (Wood et al., 2017), this study works to begin identifying determinants for core components of multilevel service delivery frameworks.

### Purpose of this Paper and Rationale for Selected Frameworks

There are two primary aims for this paper. First, we draw from exemplar multilevel service delivery frameworks in child mental health service settings to propose a conceptual foundation and identify core components. Second, with a goal of identifying commonalities across frameworks and service settings, we highlight prominent implementation determinants for each core component. Our overarching goal for this project is to facilitate knowledge sharing between researchers, system leaders, and implementation intermediaries/practitioners who may focus most explicitly on one service system but would benefit from a broader knowledge base. Our preliminary analysis can be built upon in future research that identifies how implementation determinants affect implementation outcomes, which can ultimately inform identification and testing of tailored implementation strategies.

In selecting the multi-level service delivery frameworks that served as the foundation for this paper, the author team developed inclusion criteria to assist in identifying a central and representative model used in each of the settings in which youth most commonly access mental health care—schools, outpatient settings, and primary care (Duong et al., 2021). Specifically, inclusion criteria prescribed that each model should: (a) address the full range of youth mental health needs through services provided at multiple levels of intensity (i.e., via prevention, early intervention, and treatment), (b) have empirical support, and (c) have research demonstrating the widespread adoption of the model within the given service sector (e.g., based on the number of agencies implementing, support of framework in state or federal policies, etc.). These criteria were developed organically at the outset of this paper’s conceptualization and the review process guided by expert consensus and literature focused on each framework. For criterion A, the author team discussed all possible multi-level frameworks in the three mental health service settings. In some cases, one framework emerged as the best exemplar for that particular setting (i.e., CoCM and SoC) based on consensus opinion, empirical research demonstrating efficacy (e.g., Asarnow et al., 2015; Kolko et al., 2014), and widespread adoption of the framework (e.g., Graaf et al., 2021; Kutash et al., 2011). For other settings (i.e., schools), several multi-level frameworks were considered (e.g., Interconnected Systems Framework [ISF], Multitiered Systems of Support-Behavior [MTSS-B]) and discussed relative to the criteria before consensus was reached. For criteria B and C, the authors were guided by established definitions for evidence-based interventions (e.g., What Works Clearinghouse) and reference to widespread adoption of the framework as outlined in either federal and/or state policies or empirical studies (e.g., Asarnow et al., 2015; Bradshaw et al., 2012; Miller et al., 2012; Pas & Bradshaw, 2012; Solberg et al., 2013).

Within the educational setting, positive behavioral interventions and supports (PBIS; Horner & Sugai, 2015) was selected given its recognition as one of the most widely adopted evidence-based frameworks in schools and districts. Adopted in over 25,000 schools (Center on PBIS, 2023a), PBIS is a three-tiered prevention framework associated with reductions in students’ problematic behaviors and improvements in prosocial behavior (e.g., Bradshaw et al., 2010; McIntosh et al., 2011). Next, the collaborative care model (CoCM), which is a service model for integrating mental and behavioral health services into primary care, was selected (Goodrich et al., 2013; Richardson et al., 2017). CoCM has been studied in over 80 randomized controlled trials (AIMS Center, 2023), and meta-analyses have shown that CoCM is effective in bringing about positive mental health outcomes (e.g., Asarnow et al., 2015). Within community mental health settings, system of care (SoC; Stroul et al., 2008, 2021)—a framework to support youth with significant mental health concerns (Stroul & Friedman, 1986)—was selected on the basis of its support through state and federal policy (Center for Mental Health Services, Substance Abuse and Mental Health Services Administration, and U.S. Department of Health and Human Services, 2015) and association with improvements in youth emotional and behavioral health and effective use of services (Graaf et al., 2021; Stroul et al., 2021). In Table 1, we provide an overview of each of these frameworks, including service setting, a brief description, key contributors/collaborators, applications, and a summary of the evidence-base.

**Table 1:** Exemplary multilevel service delivery frameworks in child mental health service settings

Characteristic	Exemplar framework		
	Collaborative care (CoCM)	Positive behavioral interventions and supports (PBIS)	System of care (SoC)
Setting	Primary care	Education	Community-based mental health
Description	CoCM is a service model for integrating mental and behavioral health services into primary care. A multidisciplinary team—most often including a general practitioner or physician, mental health specialist, and a designated care coordinator—work together to coordinate population-based, client-centered care (e.g., Katon et al., 2010; Lyon et al., 2016; Unützer et al., 2013).	PBIS is a three-tiered framework used in education to improve and integrate the data, systems, and practices affecting student outcomes on a schoolwide basis. Specifically, PBIS is a population-based approach to prevention in schools that involves the delivery of evidence-based services along a continuum based on student need (Jimerson et al., 2016; McIntosh and Goodman, 2016).	SoC is a framework targeted to support youth with significant mental health concerns in community mental health settings (Stroul & Friedman; 1986). The framework focuses on (1) a comprehensive array of services and supports (e.g., care coordination via wraparound; Walker et al., 2008), (2) structures and processes to facilitate service provision, and (3) a philosophy of service delivery that is youth and caregiver- driven and prioritizes culturally/linguistically responsive care (Stroul et al., 2021).
Key contributors and collaborators	General practitioner or primary care physician; mental health specialists, such as a psychiatrist, psychologist, psychiatric nurse, or social worker; care manager	Family members, students, educators (e.g., teachers or other direct implementers), administrators and board members (school, district, region and state), and community partners and agency representatives and partners	Youth with behavioral health needs and their families, community-based mental health providers, child protection, juvenile justice, school personnel, and natural supports (e.g., extended family, church, neighbors, mentors)
Applications	Although originally developed for and extensively used with adult populations, CoCM has been applied across a variety of youth mental health challenges, including behavior problems, attention deficit hyperactivity disorder (ADHD), and anxiety in children (e.g., Kolko et al., 2010; Kolko et al., 2014), as well as depression in adolescents (e.g., Asarnow et al., 2005; Richardson et al., 2014).	PBIS is typically applied on a schoolwide basis to deliver practices and intervention systems that create positive, predictable, equitable and safe learning environments and enhance students' academic and behavioral outcomes (Center on PBIS, 2015; McIntosh & Goodman, 2016). PBIS can be integrated with other multi-tiered practices and systems focused on academics, such as Response to Intervention (RtI; Ikeda et al., 1996), and student mental health (e.g., Interconnected Systems Framework, ISF; Splett et al., 2017).	SoC has been applied and interpreted across U.S. regions, populations, area of emphasis, and child-serving settings (e.g., mental health, child welfare; Stroul et al., 2015). SoC is the foundation for the Federal children’s mental health initiative (Stroul et al., 2010), which continues to provide resources to expand and sustain SoC nationwide via the Substance Abuse and Mental Health Services Administration (SAMHSA, 2020) Center for Mental Health Services.
Evidence	Findings from systematic reviews and meta-analyses have shown that CoCM is more effective than usual care in achieving positive mental health treatment outcomes (e.g., Archer et al., 2012; Asarnow et al., 2015; Richardson et al., 2017; Thota et al., 2012; Woltman et al., 2012). Research supports CoCM’s cost-effectiveness (Wright et al., 2016) and care cost savings potential (Yu et al., 2017).	Research has found that PBIS results in reductions in student problem behavior (Bradshaw et al., 2010; Flannery et al., 2014) and improved prosocial behavior (McIntosh et al., 2011), academic achievement (Horner et al., 2009; Kelm et al., 2014), perceptions of school safety (Horner et al., 2009) and school organization (Bradshaw et al., 2009; Ross et al., 2011).	SoC is effective in improving youth emotional and behavioral health, community and school functioning, and effective use of services, as well as in reducing caregiver strain (Stroul et al., 2021); Suter & Bruns, 2009). At the service systems and policy levels, SoC improves interagency collaboration, increases utilization of community supports, decreases use of institutional level supports, and yields cost savings (Stroul et al., 2015)

## Core Components of Exemplar Models

In synthesizing the literature describing the evidence and implementation of each exemplar model, we identified core components of multilevel service delivery frameworks via a multi-stage review and analysis process. The research team that undertook this work was composed of individuals with expertise in youth mental health services in one or more service delivery settings and implementation science. During the first stage, the team completed a review of representative literature for each framework, specifically relating to its evidence base, key features, and implementation considerations. At least two members of the team completed the review for each framework and developed syntheses regarding the given framework’s guiding principles, key features, and discrete practices. Written syntheses were refined through discussion within pairs, as applicable. Then, the larger team engaged in group discussion to identify commonalities across frameworks and to reach consensus on core components that spanned each exemplar framework. This discussion centered the distillation of each framework’s guiding principles, key features, and discrete practices, which were compared, contrasted, and labeled. Our goal was to identify frameworks’ shared core components, recognizing that each framework may have additional unique features that may not be representative of the essential elements of another framework. The identified core components were revised and refined through additional discussion, which centered the labels applied to core components, potential areas of overlap, and areas for consolidation. We revisited the reviewed literature or technical assistance documentation during this process. Finally, we identified illustrative examples of each component as applied to each framework, and made additional revisions to component labels or definitions, as indicated.

The resulting core components included the following: a) a systems-level approach, b) data-driven problem solving and decision-making, c) multiple levels of service intensity using evidence-based practices (EBPs), d) cross-linking service sectors, e) multiple providers^1^ working together, including in teams, and f) built-in implementation strategies that facilitate delivery of the overall model. Consistent with the multilevel nature of these service delivery frameworks, the identified core components also include multilevel features (e.g., interventions provided at varied service levels/intensity, data collected at multiple levels [client, system], teams comprised of individuals who work at varied levels within their service setting/organization, etc.). Each core component is summarized in Table 2 and described below.

**Table 2:** Core components of multilevel service delivery frameworks and examples illustrated for exemplar frameworks

*Core component* and definition	Exemplar framework		
	Collaborative care (CoCM)	Positive behavior interventions and supports (PBIS)	System of care (SoC)
*Systems-level approach to organization and service delivery* Structures and/or processes that enable service coordination within broader sociopolitical systems and engagement of actors across local, regional, and state policy, administrative, and provider levels of a system in order for the framework to be adopted/ implemented with high quality	Strategic redesign of service delivery is often needed to successfully integrate and deliver CoCM Emphasizes population-based care for a defined group of patients CoCM structures supported under new Medicaid reimbursement rules	Alignment of policies and procedures across classroom, grade, building, district, and state levels Emphasizes a whole school/district approach, with involvement from all individuals in both classroom and non-classroom settings in a school building Implementation of EBPs, systems, and associated data-based decision-making are adapted to the context of the local culture such that characteristics and cultural learning histories of contributors/collaborators, implementers, and consumers are embedded in a comprehensive and authentic manner	Requires action at the local, regional, and state levels, including to support cross-sector care coordination and service provision at the youth- and family-level Practices for systems-level management and coordination Incorporates elements of a population-based public health framework (promotion, prevention, screening, early identification and intervention, in addition to treatment) Linkages between child-serving agencies and programs across administrative and funding boundaries
*Data-driven problem solving and decision-making* Data, about clients or service delivery, are used to guide delivery of component Interventions and implementation and evaluation of frameworks	Measurement based care (i.e., treatment goals set to measurable targets, data used to monitor patient progress) Relies upon validated instruments	Ongoing facilitation and use of a problem-solving process to support planning, implementing, and evaluating effectiveness of services Comprehensive, efficient, and user-friendly data systems for supporting decision-making at all levels from the individual student level up to the aggregate district level Continuous progress monitoring Universal & comprehensive screening to inform service delivery Fidelity of implementation measures	Use of a problem-solving process to support planning, implementing, and evaluating effectiveness of services Outcome-based service provision Family and youth benchmarks of functioning Fidelity of implementation measures
*Multiple levels of service intensity using evidence-based practices (EBP)* A public health approach to service delivery in which interventions are provided to youth/families at increasing levels of intensity (i.e., promotion, prevention, early intervention, intensive treatment) and using research-based practices	Screening, evaluation, and progress-monitoring data are used to guide decisions about level of stepped care; referrals provided to specialty care, social services, and community-based resources as needed Focus from prevention to referral for intensive treatment Intensity of treatment is augmented based on client response and in accordance with progress-monitoring data	Continuum of EBP Includes supports at Tier 1 (universal), Tier 2, and Tier 3 An integrated and sequenced organization of practices is developed such that (a) a core curriculum is provided for all students, (b) modification of this core is arranged for students whose performance identified as nonresponsive, and (c) specialized and intensive curriculum is developed for students whose performance is deemed nonresponsive to the modified core Elements of this continuum have empirical evidence to support efficacy, effectiveness, relevance and social validity, and durability	Cross-sector systems integration and care coordination at the community level 3 levels of service delivery (primary and secondary prevention, tertiary intervention) Intensive family and youth-driven wraparound teams Customized, individualized service delivery All relying upon evidence-informed and promising practices, as well as interventions supported by practice-based evidence
*Cross-linking service sectors* Professionals and resources from at least two service sectors (e.g., physical health and mental health) are coordinated for service provision	Physical health & mental health care Referrals made to other sectors (specialty care, behavioral health, social services), as indicated by client need	Education and mental health (e.g., wraparound supports and culturally responsive person-centered planning that actively involves family and community supports and resources)	Key feature of SoC Single plan of care per youth/family that is linked across children’s mental health, child protection, juvenile justice, education, and community support service sectors Linkages across administrative and funding boundaries
*Teaming/multiple providers working together* Multiple providers with varied expertise work together to coordinate services (e.g., collaboratively developed treatment plans) in support of youth/family outcomes	Physical and mental health providers co-located Clearly defined roles for each care team member Collaboratively develop and share treatment plans that incorporate patient goals Regular team meetings	Implementation of EBPs and systems are guided, coordinated, and administered by a local team comprised of representation from leadership, implementers, consumers (i.e., youth, families, peers), and content experts Team ensures high intervention fidelity, management of resources, and data-based decision-making Local personnel have high levels of content knowledge, fluency, and experience to support the culturally relevant and high-intervention-fidelity of EBPs and systems	Providers brought together at the youth- and family-level through wraparound; create an individualized service plan in partnership with child and family Care management at the practice level to ensure coordination of services, and modifications based on changing needs Community and state-level cross-sector collaboratives develop cross-system agreements and financing Family and Youth Peer Support components that require workforce development unique to SoC
*Built-in Implementation Strategies*			
Examples of implementation strategies ^a^ included as core components of the service delivery frameworks			
Train and educate stakeholders	✓ Conduct ongoing training, provide ongoing consultation (administrative, clinical)	✓Conduct ongoing training, provide ongoing consultation (coaching)	✓ Conduct ongoing training (workforce training), provide ongoing consultation (coaching)
Provide interactive assistance	✓ Centralize technical assistance (e.g., AIMS Center), Provide clinical supervision	✓ Facilitation/problem-solving, Provide local technical assistance, Centralize technical assistance (e.g., Center on PBIS)	✓ Provide local technical assistance, Centralize technical assistance (e.g., state-level)
Support clinicians	✓ Revise professional roles, Create new clinical teams		
Engage consumers	✓ Prepare patients/consumers to be active participants (client engagement via education, brief interventions, motivational support, linkages with community resources)		✓ Increase demand (e.g., via strategic communication, outreach, advocacy)
Change infrastructure	✓ Change record systems (clinical information/data systems)	✓ Change record systems (clinical information/data systems), Develop local policy that supports implementation (alignment)	
Use financial strategies	✓ Place innovation on fee for service lists/formularies (Medicaid)		✓ Access new funding (cross-sector funding linkages), Place innovation on fee for service lists/formularies (Medicaid)
Adapt and tailor to context	✓ Promote adaptability (adapt treatment if targets are not reached)		
Use evaluative and iterative strategies	✓ Purposely reexamine the implementation (review provider and program outcomes to inform quality improvement actions)	✓ Assess for readiness and identify barriers and facilitators (emphasizes team-based readiness for implementation), Develop a formal implementation blueprint, Develop and organize quality monitoring system (fidelity monitoring)	✓ Develop and organize quality monitoring system (fidelity, quality, effectiveness, outcomes)
Selected references for further reading	Campo et al. (2005), Goodrich et al. (2013), Kolko and Perrin (2014)	McIntosh and Goodman (2016), Center on PBIS (2015)	Pires (2010), Stroul et al. (2010), Stroul et al. (2021)

*EBP* evidence-based practice

^a^To facilitate consistency in language (Proctor et al., 2013), implementation strategy examples are organized by categories (Waltz et al., 2015) and names articulated in the Expert Recommendations for Implementing Change (ERIC) study (Powell et al., 2015) and School Implementation Strategies, Translating ERIC Resources (SISTER) project (Cook et al., 2019; i.e., for PBIS, when applicable)

### Systems-Level Approach to Organization and Service Delivery

Integral to each framework is a systems-level approach to the organization and delivery of mental health care to youth clients and their families. The systems-level approach describes structures and/or processes that enable service coordination within larger sociopolitical systems, enacting or clarifying policy at varied levels, and involvement and coordination across invested actors within a given service setting. As such, multilevel frameworks require engagement of individuals across levels within a system, including state and local policy makers, administrative decision makers, direct service providers (e.g., school, practice, behavioral health agency), community members, clients, and families, and consider the influence of social environments on human behavior. Drawing from bioecological systems theory (Bronfenbrenner & Ceci, 1994), a systems-level approach emphasizes that children are shaped by their interaction with others and their contexts (e.g., family, peers, connections between providers and family, state/federal policies related to mental health). Therefore, effectively delivering services within each framework requires augmenting organization and/or service-setting-level priorities, administrative workflow, and decision-making, as well as shaping provider and client interactions. For example, CoCM necessitates the strategic redesign of service delivery such that mental health care is integrated into primary care practice teams, data-systems, treatment planning and delivery, and provider accountability metrics (Goodrich et al., 2013). SoC requires integration of services within a behavioral health system and specifies practices for system-level management, coordination, and integrated care management (Stroul et al., 2010). Supportive infrastructure at the local, regional, and state levels enables cross-sector care coordination and service provision (Stroul et al., 2021). Each framework further prioritizes a population-based or public-health approach to service delivery, whether for a defined group of patients (CoCM), a whole school or district (PBIS), or children and families in the local community (SoC).

### Data-Driven Problem-Solving and Decision-Making

As approaches to organizing and structuring a spectrum of circumscribed practices, data-driven problem solving and decision-making processes within multilevel frameworks have two primary purposes. First, client data are collected to guide prevention and treatment decisions. Second, implementation and service process and/or outcome data are collected to inform ongoing implementation and evaluation of the component interventions and the frameworks themselves. Client data collection to inform service delivery may include population-level mental health screening, diagnostic assessment (e.g., for those referred via screening), and intervention progress monitoring (Campo et al., 2005; Molloy et al., 2013; Stroul et al., 2021). Implementation of each framework may be guided by fidelity data (i.e., intervention and strategy) and/or service delivery metrics (e.g., number of youths screened). In CoCM, for example, physicians may administer mental health screenings and refer youth to mental health specialists for further evaluation and triage. Clinical data then inform development of treatment protocols for each client. Treatment goals are aligned toward measurable targets and treatment is augmented based on progress-monitoring data (e.g., Goodrich et al., 2013; Lyon et al., 2016). Clinical information systems (e.g., electronic health records) and review of population-level data facilitate monitoring of care provision at the practice-level and evaluation of provider- and program-level outcomes to inform quality improvement (AIMS Center, 2014). Similarly, intervention fidelity data collected using instruments designed for PBIS (e.g., Tiered Fidelity Inventory) facilitates school teams’ problem-solving processes that support planning, implementation, and evaluation of tiered services (Algozzine et al., 2019). Readers are referred to Bickman et al. (2016) for a broader discussion of how data may be used to facilitate precision in mental health treatment.

### Multiple Levels of Service Intensity Using EBPs

Within each exemplar multilevel service delivery framework, services are provided to youth and/or families at increasing levels of intensity. Each framework focuses on prevention, early identification and intervention, and intensive treatment, with providers using EBPs at each level to prevent or address known problems. For example, within PBIS, a continuum of EBPs—from universal (all students and staff in all settings) to targeted (some students) to intensive (few students with greatest need)—are layered across the school and grade/classroom levels (Center on PBIS, 2015). When implemented well, PBIS prioritizes prevention via high quality learning environments, including universal behavioral expectations and social-emotional learning and supports, for all students and staff and across all settings (i.e., school-wide, classroom, and non-classroom settings; Center on PBIS, 2015). Targeted and intensive interventions are focused, frequent, and small group or individually oriented, respectively. These interventions are provided in addition to universal interventions, with a goal of reducing the number and/or intensity of students’ behavioral or social-emotional challenges (Center on PBIS, 2015).

### Cross-Linking Service Sectors

Effectively changing systems to support data-based decision-making and EBP across multiple service tiers is dependent upon the cross-linking of service sectors and multiple providers with varied expertise working together within the local context. These two core components may be clearly differentiated for some exemplar multilevel service delivery frameworks (i.e., CoCM and SoC) which involve co-location and care coordination across service systems, but are less differentiated within other frameworks (e.g., PBIS). While we discuss each component separately, their interdependent nature is essential to keep in mind. Cross-linking of service sectors implies that professionals and resources from at least two service sectors are coordinated for service delivery. For example, within SoC, a single plan of care per family is developed, with care coordinated across sectors, including children’s mental health services, child protection, juvenile justice, education, and community support sectors. This systems-level integration includes the creation of linkages between child-serving agencies and programs across administrative and funding boundaries (Stroul et al., 2010). This might be achieved by creating a “bundled” payment system that allows for enhanced care coordination, contracted services, and flexible spending. As an example, New Hampshire established a comprehensive Medicaid benefit (via 1951[i] mechanism; 2018), that supports the intensive wraparound model. Cross-sector linkages in SoC are further supported through SoC teams that discuss policy, systems, and practice-level changes, and that prioritize collaboration.

### Multiple Providers Work Together

Within each framework, multiple providers with varied expertise work together intentionally to support youth and family outcomes, for example, via practice, care, or problem-solving teams. Common across each model are team-developed prevention, treatment or outcome plans with goals that are collaboratively generated. Multidisciplinary CoCM teams (e.g., primary care practitioner, mental health specialist, and care manager) include each team member operating within a clearly defined role (Campo et al., 2005; Lyon et al., 2016). For example, physicians may screen for mental health symptoms and make referrals to specialists who provide further evaluation whereas care managers may act as liaisons, provide brief intervention, and monitor clients’ progress (e.g., Campo et al., 2005; Katon et al., 2010). PBIS teams include general educators, special educators, school mental health professionals and administrators who leverage collected data and formal problem-solving procedures to monitor and enhance implementation (McIntosh & Goodman, 2016). In SoC, effective wraparound practice occurs within a coordinated network led by the expressed needs and goals of the youth and family, including the care coordinator, supervisor/coach, administrators, peer supporters, community-based providers, and systems level partners (e.g., regional/state-level child protection authority), who each have specialized knowledge/skills (Miles et al., 2019; Schurer et al., 2016; Walker et al., 2008).

### Built-In Implementation Strategies

Each multilevel framework also includes several practices, tools, or techniques—implementation strategies—that facilitate agencies’ and/or contributors’ implementation of the overall framework. Unlike stand-alone EBP, many implementation strategies tend to be “baked into” multilevel service delivery frameworks. Prominent examples of implementation strategies that are core within each framework are included in Table 2, with strategies organized by category and labeled with names provided in the Expert Recommendations for Implementing Change (ERIC) study (Powell et al., 2015; Waltz et al., 2015) or School Implementation Strategies, Translating ERIC Resources (SISTER) project (Cook et al., 2019).

A variety of discrete strategies, applied with specific service settings in mind, are represented across categories. For example, *training and education* of respective contributors through initial and ongoing training/professional development and/or consultation/coaching in the target framework is prioritized (George et al., 2018; Kolko et al., 2014; Stroul et al., 2021). Strategies broadly characterized by *providing interactive assistance* are also prominent and include clinical supervision in CoCM (e.g., case managers with psychiatrists), as well as regional, state, or national-level technical assistance (TA) centers for each framework (e.g., Advancing Integrated Mental Health Solutions [AIMS] Center for CoCM, National Center on PBIS, state/regional TA in SoC; Center on PBIS, 2015; Stroul et al., 2021). Congruent with an emphasis on using data to guide decision-making, CoCM and PBIS emphasize changing record systems (*change infrastructure*), for example by modifying or implementing clinical/data information systems (e.g., Schoolwide Information System [SWIS]; Campo et al., 2005; PBISApps, 2022). *Financial strategies*, such as pursuing Medicaid reimbursement for services are often leveraged for CoCM (Goodrich et al., 2013; Wolk et al., 2021) and SoC (Stroul et al., 2021); however, we did not observe a similar focus within PBIS literature and documentation. This discrepancy may reflect differences in the financial structures of primary care and outpatient/specialty mental health settings in comparison to educational settings, such that some reimbursement models may not recognize schools as mental health services sites (Hoover & Bostic, 2021; see Schultz et al., 2020 for policy recommendations). Finally, whereas *evaluative and iterative strategies* appear to be integrated within each framework, the specific strategies prioritized may vary depending on the framework and service setting. Systems for implementation quality monitoring are common across frameworks (Goodrich et al., 2013; Stroul et al., 2021), however, PBIS implementation guidance (Center on PBIS, 2015) emphasizes conducting needs assessments and developing a formal implementation blueprint prior to implementation. As described in the discussion, much additional work is indicated surrounding the identification, specification, and tailoring of the implementation strategies that tend to be most commonly integrated into multilevel frameworks in mental health.

## Implementation Determinants for Core Components of Multilevel Service Delivery Frameworks

Although identifying core components of multilevel service delivery frameworks is important in assessing both intervention and implementation strategy fidelity (e.g., Lawson et al., 2018), research also indicates that the determinants that affect implementation of each component may vary (e.g., Wood et al., 2017). Identifying how implementation determinants interface with frameworks’ core components and similarities in the determinants that are relevant across frameworks can facilitate future research that seeks to evaluate the impact of determinants on implementation outcomes as well as the identification, specification/tailoring, and testing of implementation strategies tailored to these determinants.

Therefore, after identifying the core components of each exemplar model, we sought to map relevant implementation determinants, delineated in the implementation literature, to each multilevel service delivery framework component. We repeated the multistage review and analysis process described above with emphasis on implementation of each exemplar framework. Reviewed literature included empirical studies evaluating a given framework, so long as implementation was also addressed (e.g., evaluation or reporting of implementation outcomes, process evaluation describing implementation procedures and/or challenges and successes); implementation studies (e.g., that identified or investigated implementation determinants or strategies; implementation evaluations); and systematic review articles. Across reviewed literature, the identified determinants originated from study data or were extracted from the manuscript text (e.g., results section, discussion section).

Identified determinants were subsequently coded using a determinant framework (Nilsen, 2015)—the Consolidated Framework for Implementation Research (CFIR; Damschroder et al., 2009). The CFIR is a widely-used, meta-theoretical framework that delineates a set of constructs, with definitions, that may influence outcomes of implementation efforts across settings (Damschroder et al., 2009, 2022). Thus, the CFIR provides a structure for synthesizing determinants across studies and settings using a common terminology (Damschroder et al., 2009; Kirk et al., 2020). Four CFIR domains were of focus, including: the outer setting (e.g., policies and incentives, inter-organizational networks), inner setting (e.g., intra-organization networks and communication, leadership, access to information), individual (e.g., knowledge and beliefs, self-efficacy), and intervention (e.g., complexity, cost) levels. For the purposes of this study, we defined the outer setting as the macro-economic, sociopolitical, and service-system context (e.g., state or local education agency, health care system); the inner setting as the more proximal features of a given service setting (e.g., school, practice); and the intervention level as encompassing framework-specific features.

Coding was led by the first author, who used the published definitions of CFIR constructs, supplementary material, and inclusionary and exclusionary criteria described in the CFIR codebook (CFIR, 2023; Damschroder et al., 2009) to determine code labels. The preliminary codes were reviewed by the remaining authors, who each hold expertise in exemplar service delivery framework(s) and/or implementation science. Determinants were recoded as indicated by feedback throughout the coding process. Aligned with our stated goals for this study, we aimed to highlight implementation determinants that were evident across multiple sources for each exemplar framework so as to be a catalyst for future work that empirically examines how relevant determinants impact implementation outcomes as well as to inform selection of context-specific implementation strategies (beyond those that are baked-in) to improve these frameworks’ outcomes (Powell et al., 2019). Thus, this process was not intended to be systematic nor exhaustive; a comprehensive review was beyond the scope of this paper but could be an important subsequent step (see Limitations and Future Directions). In the sections below and in Table 3, we present select examples of implementation determinants, organized by their relation to each core component presented previously. Determinants were identified at each contextual level.

**Table 3:** Selected implementation determinants for core components of exemplar multilevel service delivery frameworks and relation to CFIR domains and constructs

Core component	Exemplar framework		
	Collaborative care (CoCM)	Positive behavioral interventions and supports (PBIS)	System of care (SoC)
Systems level approach	Financial barriers (e.g., reimbursement challenges; Solberg et al., 2013; Sanchez et al., 2010)—**Intervention**^**a**^** (Cost), Outer Setting (External Policies & Incentives)**	Funding (Kincaid et al., 2007) and financial resources (Fox et al., 2022)—**Intervention (Cost), Outer Setting (External Policies & Incentives)**	Financial support following grant periods (Vinson et al., 2001)—**Intervention (Cost), Outer Setting (External Policies & Incentives)**
	Leadership support (i.e., strong organizational leadership; Whitebird et al., 2014)—**Inner Setting (Leadership Engagement)**	School administrator (e.g., principal) support (Chitiyo & Wheeler, 2009; Flannery et al., 2009); effective and supportive leadership (Fox et al., 2022)—**Inner Setting (Leadership Engagement)**	Strong/committed and consistent leadership support (Stroul, 2013; Stroul & Manteuffel, 2007)—**Inner Setting (Leadership Engagement)**
	Full-time staff dedicated to implementation (Wolk et al., 2021)—**Inner Setting (Available Resources, Access to Knowledge and Information)**	Receiving and maintaining support from school faculty and staff (Flannery et al., 2009); Staff beliefs that conflict with PBIS philosophy or lack of buy in, limited to commitment to framework (Fox et al., 2022)—**Characteristics of Individuals (Knowledge and Beliefs about the Intervention)**	Shared vision among contributors /leadership team (e.g., about systems change goals; Behar & Hydaker, 2009; Heflinger, 1996; Mendenhall & Frauenholtz, 2014)—**Inner Setting (Relative Priority)**
		District support (Kincaid et al., 2007)—**Outer Setting (External Policies & Incentives)**	Central entity/organization that can connect service providers/agencies (Heflinger, 1996; Vinson et al., 2001)—**Inner Setting (Structural Characteristics)**
		Opportunities for quality team and staff trainings (Chitiyo & Wheeler, 2009; Kincaid et al., 2007; Flannery et al., 2009)—**Inner Setting (Available Resources)**	Commitment, support, priority from macro/higher-level policy and decision makers at state and local levels (e.g., to provide funding, express priority, support compliance, provide funding; Behar & Hydaker, 2009; Lunn et al., 2011; Stroul, 2013; Stroul & Manteuffel, 2007)—**Outer Setting (External Policies & Incentives)**
		Multiple schools within a district implementing PBIS with fidelity and number of schools beginning implementation at the same time (Fox et al., 2022)—**Outer Setting (Cosmopolitanism)**	
		Lack of access to needed resources (Fox et al., 2022)—**Inner Setting (Available Resources)**	
Data-driven problem solving and decision-making	Limited familiarity/understanding of how to implement this component of CoCM (Wood et al., 2017)—**Inner Setting (Readiness for Implementation, Access to Information and Knowledge), Individual (Knowledge and Beliefs about the Intervention)**	Administrator support and input (Chitiyo & Wheeler, 2009; McIntosh et al., 2013)—**Inner Setting (Readiness for Implementation, Leadership Engagement)**	Limitations in data available that can support decision-making or implementation evaluations (Stroul, 2013; Suter & Bruns, 2009 )—**Inner Setting (Readiness for Implementation, Available Resources)**
	Challenges with data/information systems (e.g., building infrastructure, integrating into practice, duplication, access limitations, inefficiency; Overbeck et al., 2016; Wolk et al., 2021)—**Inner Setting (Readiness for Implementation,Available Resources)**	Effective collection and use of data (e.g., implementation/service or client outcome data), including sharing data within and outside the school (Fox et al., 2022; Kincaid et al., 2007; Mcintosh et al., 2013) and frequency of data sharing with whole school (McIntosh et al., 2015)—**Inner Setting (Readiness for Implementation, Available Resources)**	Understanding importance of evaluation and data in driving services (Behar & Hydaker, 2009)—**Inner Setting (Access to Knowledge and Information)**
	Absent progress monitoring procedures (Overbeck et al., 2016)—**Intervention (Design Quality and Packaging), Inner Setting (Compatibility)**	Regularly sharing data regarding implementation and its effects (Flannery et al., 2009)—**Process (Reflecting & Evaluating)**	Methods for sharing data in real-time about system trends and to inform data-based decision-making (Behar & Hydaker, 2009)—**Inner Setting (Readiness for Implementation, Available Resources)**
	Staff knowledge (understanding) and confidence regarding intervention expectations, data collection/screening procedures, etc. (Wood et al., 2017)—**Intervention (Design quality and packaging, Complexity), Inner Setting (Readiness for implementation, Access to information and knowledge), Individual (Knowledge and beliefs about the intervention)**	Staff buy-in (Chitiyo & Wheeler, 2009; McIntosh et al., 2013)—**Characteristics of Individuals (Knowledge and Beliefs about the Intervention)**	
	Screening connected to standardized care pathway, inclusive of high-quality materials and training (Wood et al., 2017)—**Intervention (Design Quality and Packaging), Inner Setting (Access to Knowledge and Information)**		
	Quality of data collection tools (Wood et al., 2017)—**Intervention (Design Quality and Packaging, Evidence Strength and Quality)**		
Multiple levels of service intensity using evidence-based practice	Systematic monitoring of patients and follow-up (i.e., access to/availability of information, or efforts to set up; Overbeck et al., 2016; Wood et al., 2017)—**Inner Setting (Readiness for Implementation, Access to Knowledge and Information, Compatibility)**	Resources, including time and for training, to support interventions, particularly at Tier 3 (Fox et al., 2022)—**Inner Setting (Available Resources)**	Difficulty establishing shared service plans (i.e., due to challenges with cross-sector teams/collaboration; Mendenhall & Frauenholtz, 2014; Stroul, 2013)—**Inner Setting (Networks and Communications), Intervention (Complexity)**
	Limited referral sites for patient's requiring referral due to high level of need (Wolk et al., 2021)—**Inner Setting (Readiness for Implementation, Available Resources), Outer Setting (Cosmopolitanism)**	Individualizing interventions at each service level, for example, using more behavior management tools during intervention (Chitiyo & Wheeler, 2009)—**Intervention (Adaptability)**	Lack of professionals with training to provide specific types of services within the full service array (Heflinger, 1996)—**Inner Setting (Readiness for Implementation; Available Resources)**
			Lack of understanding of SoC approach and how to put it into practice, why important to change current practices to provide full service array (Stroul, 2013)—**Intervention (Relative Advantage), Inner Setting (Access to Knowledge and Information)**
Cross-linking service sectors	Physician/physical health provider discomfort with diagnosing/treating mental health problems (psychiatric treatment; Overbeck et al., 2016; Wolk et al., 2021)—**Inner Setting (Readiness for implementation, Access to Information and Knowledge), Individuals (Self-Efficacy)**	Prioritization/alignment with current approaches (Fox et al., 2022)—**Inner Setting (Implementation Climate—Compatibility)**	Differences in service philosophy across agencies/agencies' limited understanding of one another's role, mandates, scope of services, service limitations all of which can challenge communication and relationships, and create conflict (Heflinger , 1996; Mendenhall & Frauenholtz, 2014; Stroul, 2013)—**Outer Setting (Cosmopolitanism), Inner Setting (Networks and Communications)**
	Co-location of providers from different service sectors (Wood et al., 2017)—**Inner Setting (Structural Characteristics, Networks and Communications), Outer Setting (Cosmopolitanism)**	Use of experts from within and outside of the school to offer training and explain PBIS benefits (Flannery et al., 2009)—**Inner Setting (Available Resources), Outer Setting (Cosmopolitanism)**	Insufficient commitment across child-serving systems (i.e., some agencies being more difficult to engage or are less willing to share resources; Stroul, 2013)—**Inner Setting (Relative Priority)**
	Integrated information systems that facilitate sharing of notes & messages (Wood et al., 2017); potential concerns about physician autonomy and data privacy (Overbeck et al., 2016)—**Inner Setting (Networks and Communications, Compatibility)**		Shared values among partners (Behar & Hydaker, 2009)—**Inner Setting (Relative Priority)**
	Provider shortages (Sanchez et al., 2010)—**Inner Setting (Access to Knowledge and Information, Available Resources)**		Entities work collaboratively (Behar & Hydaker, 2009)—**Outer Setting (Cosmopolitanism)**
Teaming/multiple providers working together	Breakdowns in networks and communication pathways (e.g., avoiding communication, using jargon, limited technology to support timely communication; Wood et al., 2017); Enhanced inter-provider communication, integrated information systems multidisciplinary meetings (Wolk et al., 2021; Wood et al., 2017)—**Inner Setting (Networks and Communications)**	Effective team communication and use of team meetings; team composition is representative (Fox et al., 2022); Issues related to team functioning and communication (Kincaid et al.; 2007), including lack of consistency (Chitiyo & Wheeler, 2009)—**Inner Setting (Networks and Communications)**	Formalizing communication (Vinson et al., 2001) and communication challenges (e.g., decision-making conversations not inclusive of all contributors; Mendenhall & Frauenholtz, 2014)—**Inner Setting (Networks and Communication), Intervention (Complexity)**
	Staff turnover (Overbeck et al., 2016)—**Inner Setting (Structural Characteristics, Available Resources)**	High staff turnover (Fox et al., 2022)—**Inner Setting (Structural Characteristics)**	Differing priorities among contributor s and insufficient commitment across clinicians, care managers, and their organizations (Heflinger, 1996; Stroul, 2013)—**Inner Setting (Relative Priority)**
	Organizational culture and readiness for change (e.g., impacting attitudes, changes in practice, integration of mental health care, sharing care of patients; buy-in; Wood et al., 2017)—**Inner Setting (Implementation Climate, Relative Priority, Culture, Readiness for Implementation, Access to Knowledge and Information)**	Staff trainings (Chitiyo & Wheeler, 2009); Need for technical assistance in data collection and recording, soliciting administrative support, and monitoring intervention implementation (Chitiyo & Wheeler, 2009)—**Inner Setting (Available Resources)**	Co-location, facilitating active collaboration (Vinson et al., 2001)—**Inner Setting (Structural Characteristics, Networks and Communications)**
	Clearly defined roles (Wood et al., 2017); Lack of agreement about responsibility for care (Sanchez et al., 2010)—**Intervention (Complexity), Inner Setting (Culture)**	Inadequate parental support (Chitiyo & Wheeler, 2009), difficulty generating student involvement in implementation (Flannery et al., 2009)—**Inner Setting (Relative Priority)**	Strong, trusting working relationship (Behar & Hydaker, 2009)—**Inner Setting (Networks and Communication)**
	Clear leadership structure led by providers across service sector (Wood et al., 2017)—**Inner Setting (Leadership Engagement)**	Staff knowledge and awareness of relevant theories/activities (Fox et al., 2022)—**Characteristics of Individuals (Knowledge and Beliefs about the Intervention), Inner Setting (Readiness for Implementation, Access to Knowledge and Information)**	
	Co-location (Overbeck et al., 2016; Wood et al., 2017)- **Inner Setting (Structural Characteristics)**		

Bold text depicts determinant domains and constructs (the latter specified using parentheses) using the Consolidated Framework for Implementation Research (CFIR; Damschroder et al., 2009)

^a^Intervention factors indicate features related to the multilevel service delivery framework or its component parts

### Systems Level Approach

Determinants of implementation of the systems level approach, unsurprisingly, are often situated within the outer and inner settings. For example, within the outer setting, *external policies and incentives* include explicit support from higher-level policy and decision makers at state or local levels who can place priority on implementation and support compliance with enacted policies (Stroul & Manteuffel, 2007). Supportive policies that provide funding or facilitate reimbursement for component interventions may counter financial barriers to implementation (e.g., insufficient funding following grant award periods; Stroul, 2013; Vinson et al., 2001). Within the inner setting, strong organizational leadership (i.e., *leadership engagement*) can guide implementation of the multilevel service delivery framework across the system—whether that is a primary care practice, local school or school district, or community behavioral health agency (Fox et al., 2022; Stroul, 2013; Whitebird et al., 2014). Developing a shared vision and goals for implementation across implementers within the organization is also a prominent determinant of this component (i.e., *relative priority*; Heflinger, 1996; Mendenhall & Frauenholtz, 2014). Access to adequate *resources*, such as full-time staff who support implementation (Wolk et al., 2021) and frequent opportunities for quality training and ongoing technical assistance, also appear important (Flannery et al., 2009; Fox et al., 2022).

### Data-Driven Problem Solving and Decision-Making

Inner setting factors, including *readiness for implementation* and *available resources*, were similarly prominent for data-driven problem solving and decision-making. For example, providers’ use of client data to triage clients to the appropriate level of care or guide treatment decisions, and implementation/service data to evaluate implementation progress may be influenced by the extent to which data/information systems have been established and by providers’ understanding of how to use data for these purposes (e.g., Overbeck et al., 2016; Wood et al., 2017). For example, developing data systems to be able to manage and efficiently report data collected for multiple purposes (e.g., client, intervention and strategy fidelity, and service data, with multiple potential sources/measures for each type of data) is important for effectively using data to guide service delivery and implementation (e.g., Center on PBIS, 2023b). When insufficient data are available—such as limited intervention fidelity (Suter & Bruns, 2009) or outcome data (Stroul, 2013)—decision-making and evaluation of interventions may be challenging. Providers’ knowledge and access to information about data systems, including how data align with frameworks’ intervention expectations and procedures, are also salient (e.g., *access to knowledge and information;* Behar & Hydaker, 2009; Wood et al., 2017). Thus, attending to *individuals’ knowledge and beliefs* about the service delivery framework, for example by building buy in and providing training on the importance of and effective use of data to guide decisions, can be helpful (Chitiyo & Wheeler, 2009; Flannery et al., 2009; McIntosh et al., 2013; Wolk et al., 2021). Several characteristics of the frameworks themselves may also impact data-use, including how data-systems and progress-monitoring procedures are connected to other components of the framework (i.e., *design quality and packaging*), availability of reliable/valid data collection tools (i.e., *evidence strength and quality*), and the *complexity* inherent in collecting and using multiple forms of data (Center on PBIS, 2023b; Wood et al., 2017). For example, in PBIS, school teams are responsible for collecting and evaluating student outcome and fidelity (intervention and strategy) data to make decisions about school, classroom, and individual service delivery (e.g., which interventions are appropriate, whether to continue interventions, etc.). These decisions are further complicated by the need to simultaneously consider multiple sources of data to identify and act upon one of multiple plausible solutions that are appropriate for student(s) needs and a given school’s context (Chaparro et al., 2022).

### Multiple Levels of Service Intensity Using EBPs

Determinants for delivering EBPs with increasing levels of intensity included multiple dimensions of an organization’s inner setting, such as *available resources*, *readiness for implementation*, and providers’ *access to knowledge and information*. Select outer setting and intervention/framework components were also discussed, although less frequently. Seemingly beneficial to framework implementation is access to internal staff resources (i.e., inner setting; *available resources*, which contribute to *readiness for implementation*) as well as connections with external service providers (i.e., outer setting; *cosmopolitanism*—also see below) to facilitate referrals of clients with the highest levels of need (i.e., for CoCM and PBIS; Fox et al., 2022; Wolk et al., 2021) or to establish shared service plans (i.e., for SoC; Mendenhall & Frauenholtz, 2014; Stroul, 2013). However, provider shortages across care systems (Goforth et al., 2021; Health Resources and Services Administration, 2022) create barriers to offering the full service array (Heflinger, 1996). Importantly, the external (i.e., outer setting) factors contributing to the noted shortage of providers or referral sites (e.g., availability of graduate education programs/faculty to provide training, regional differences in provider availability and training opportunities, factors impacting retention, etc.; e.g., National Association of School Psychologists, 2021) were not a focus in the selected literature we reviewed. This seeming omission parallels the historically more limited focus on policy in implementation research, and thus is an important area for continued investigation (Crable et al., 2022). Finally, provider understanding of the service delivery framework and how to implement it (i.e., *access to knowledge and information*), including the degree to which the framework is perceived to be adaptable to needs of the local context and advantageous over current practice (i.e., *adaptability*), may impact whether current practices are augmented to support provision of the full service array (Chitiyo & Wheeler, 2009; Stroul, 2013).

### Cross-Linking Service Sectors

Coordination of professionals and resources across service sectors may be influenced by factors that impact intra-organization *networks and communication*, the degree to which the organization is connected to other organizations within the local community (i.e., *cosmopolitanism*), and the extent to which this type of coordination is compatible with ongoing service provision and providers’ perceptions of their role (i.e., *compatibility)*. For example, co-location of providers from different sectors (e.g., primary care and mental health in CoCM; *structural characteristics*) and integrated information systems (i.e., *networks and communications*) may facilitate collaboration across service sectors that enables service delivery (Wood et al., 2017). However, providers (e.g., primary care physicians) or service systems (e.g., schools) whose role/mission has traditionally not involved supporting child mental health may be hesitant to engage in related interventions and resistant to the collaboration required for implementation of multilevel service delivery frameworks to be successful (e.g., *self-efficacy*, *compatibility*; Fox et al., 2022; Overbeck et al., 2016; Wolk et al., 2021). Further, time spent in cross-system collaboration is typically not reimbursable. When care is coordinated across multiple agencies, such as in SoC, differences in service philosophy and gaps in understanding related to the roles and functions of partner agencies can challenge communication, strain relationships, and create conflict (e.g., *networks and communications*, *cosmopolitanism*; Heflinger, 1996; Mendenhall & Frauenholtz, 2014; Stroul, 2013). Thus, the degree to which the multilevel service delivery framework is aligned with the provider’s or agency’s larger mission and ongoing efforts is likely important for coordination (i.e., *compatibility*; Fox et al., 2022). Together, prominent determinants to effective cross-linking of service sectors suggest that co-location may be insufficient for effective care coordination, but rather that a shared vision (e.g., *relative priority*) and proximal (e.g., *networks and communications*) and distal systems (e.g., *cosmopolitanism*) to support effective communication and collaboration are also needed (Behar & Hydaker, 2009).

### Multiple Providers Work Together

The degree to which multiple providers work together (e.g., via teaming) to support youth and family outcomes may also be impacted by provider *networks and communication* pathways. When these breakdown (e.g., jargon, ineffective communication) or formal communication systems (e.g., integrated information systems) are absent, provider collaboration can be impeded (Kincaid et al., 2007; Mendenhall & Frauenholtz, 2014; Wood et al., 2017). Communication across care team members may be enhanced through integrated information systems, multidisciplinary meetings, and fostering strong working relationships (Behar & Hydaker, 2009; Wolk et al., 2021). Similarly, provider collaboration may be supported when teams are representative, members feel included, and roles and responsibilities for care are clearly defined—however, these may also be challenged by the *complexity* inherent within multilevel service delivery frameworks (Fox et al., 2022; Sanchez et al., 2010). High rates of staff turnover (i.e., *structural characteristics*; Fox et al., 2022; Overbeck et al., 2016), providers’ perceptions about the importance of multilevel service delivery framework implementation (i.e., *knowledge and beliefs about the intervention*), and shared commitment among providers, agencies, and youth/families (i.e., *relative priority*) may impact care coordination (Chitiyo & Wheeler, 2009; Flannery et al., 2009; Heflinger, 1996; Stroul, 2013). Organizational readiness for change (supported by engaged leaders; i.e., *readiness for implementation*, *leadership engagement*), provider’s access to information/support for team-based care activities (i.e., *access to knowledge and information*), and provider attitudes about engaging in these practices (i.e., *knowledge and beliefs about the intervention*) also appear relevant (Chitiyo & Wheeler, 2009; Fox et al., 2022; Wood et al., 2017).

## Discussion

Multilevel service delivery frameworks are championed as best-practice for meeting a variety of youth mental health needs and are increasingly adopted in primary youth mental health service settings (i.e., schools, primary care, outpatient/specialty mental health). Although exemplar frameworks are implemented within each service setting, their availability and quality are challenged by several factors including, but not limited to, a diffuse research base examining implementation (compared to discrete interventions). The siloed nature of the extant implementation research occurring within each setting has led to a limited understanding of the frameworks’ common core components and their respective implementation determinants, knowledge of which could promote generalization across disciplines and inform future identification of implementation strategies.

We described six core components shared by three exemplar multilevel service delivery frameworks—PBIS in schools, CoCM in primary care, and SoC in outpatient/specialty mental health settings. Multilevel service delivery frameworks require systems-level engagement that enables development of data and problem-solving systems to facilitate a graduated approach to service provision. Professionals and resources are coordinated across service sectors within an organization or community, supported by team-based care that guides service provision. Also built into these service delivery frameworks are implementation strategies that, for example, support changes to local infrastructure, emphasize training and education about these complex frameworks, and prioritize iterative evaluation and implementation.

In articulating these common essential features of child mental health service delivery frameworks, this work begins to lay a conceptual foundation for multilevel service delivery that can inform research and practice across child and adult mental health service settings. This cross-sector foundation can enable information sharing and collaboration among implementation researchers, system leaders, and/or implementation intermediaries to advance the high-quality implementation of multilevel service delivery frameworks across youth mental health service settings, which is aligned with increasing calls for collaboration across youth mental health providers (The White House, 2022). For example, technical assistance providers (e.g., Center on PBIS) and district/school-based PBIS leadership teams may learn from the successes and challenges that primary care agencies encountered with data-driven problem-solving and decision-making systems and analyze whether the approach used in primary care could be adapted to fit their local context. Practical implementation reports and research that further articulates the ways in which data-informed delivery of multilevel frameworks in each setting can facilitate this type of interdisciplinary knowledge sharing. This foundation may further support the application and testing of multilevel frameworks in novel settings, such as CoCM within schools (Lyon et al., 2016) or PBIS in juvenile justice settings (Kumm et al., 2020), and may generalize to multilevel service delivery frameworks implemented within adult mental health care settings (e.g., CoCM, behavioral health crisis care systems; Goodrich et al., 2013; SAMHSA, 2020).

Future implementation research can build upon this work. For example, future research can examine how intervention fidelity varies by core component, as well as how core components and their implementation relate to service outcomes (Blase & Fixsen, 2013). This body of work will complement research that examines the process for implementing multilevel service delivery frameworks’ core features. For example, the Stages of Implementation Completion (SIC) is a measure of implementation processes that yields information about the time spent in varied implementation activities, the proportion of activities completed, and how much of the implementation process was achieved (Saldana et al., 2011). The SIC has been adapted and applied to CoCM in rural primary care clinics and was able to accurately assess implementation effectiveness and provide information on what challenges to implementation arose and when during the implementation process (Saldana et al., 2020). Ultimately, this information can facilitate frameworks’ adoption and implementation with fidelity (i.e., intervention and implementation strategy).

### Implementation Strategies for Multilevel Frameworks

Implementation scientists recommend that implementation strategies be selected and applied to address practice- and context-specific determinants (Baker et al., 2015; Powell et al., 2017). Yet, in practice, strategies are often mismatched to determinants (e.g., Davies et al., 2010). By delineating how implementation determinants interface with core features of multilevel service delivery frameworks, this study may provide a starting point for future research that evaluates the impact of determinants on implementation outcomes, and that seeks to identify/develop, operationally define, and test tailored implementation strategies (Powell et al., 2017). We highlighted implementation determinants at multiple contextual levels that appear to be salient for multilevel service delivery frameworks. Our findings indicate that the most consequential barriers and facilitators for each framework component may vary. As such, it is likely that the strategic integration of multiple implementation strategies (“baked in” and supplemental), tailored to the multilevel determinants most salient for each framework component and context (Powell et al., 2017), is needed to advance the high-quality implementation of multilevel service delivery frameworks.

Although we identified numerous implementation strategies that are baked in as core components of multilevel service delivery frameworks, additional research is needed to systematically identify and subsequently specify how integrated strategies manifest across service models. Proctor et al. (2013) developed guidelines to promote the precise description of implementation strategies applied in research or practice, including specification of strategies’ actor, action, action target, temporality, dose, implementation outcome addressed, and justification. For example, whereas changing record systems via the development of clinical/data information systems is a key implementation strategy for CoCM and PBIS, precise descriptions of how these data information systems are developed and used—including the specific activities that are involved, in what sequence, performed by whom, and with what purpose—are needed. Specification of built in strategies as part of clinical (e.g., Moore et al., 2021; Rudd et al., 2020) and implementation research (Proctor et al., 2013), and tracking their use in implementation evaluations (Powell et al., 2019), is critical to guide the tailoring of implementation strategies for multilevel frameworks to a given service setting. Clearly identifying and specifying strategies that are built into a specific service framework may further support generalization and knowledge sharing across service contexts.

The implementation determinants that we described in this paper for each core component may serve as a preliminary guidepost in identifying strategies, in addition to those that are baked in, to support multilevel service delivery framework implementation. Although a comprehensive review of strategies that might benefit multilevel frameworks is beyond our current scope, our work suggests that coordination of implementation strategies targeting multiple contextual levels (i.e., intervention, individuals, inner and outer setting) is needed. Given that systems-level organizational change is foundational to multilevel service delivery frameworks, organization-level implementation strategies appear central to multifaceted implementation strategy design. For example, researchers and practitioners may draw upon the Leadership and Organizational Change for Implementation (LOCI) model (Aarons et al., 2015) to improve implementation leadership and develop organizational strategies to support multilevel framework implementation (see Brookman-Frazee & Stahmer, 2018 for an example adaptation study). The LOCI model includes six components (e.g., assessment, leadership training, coaching, organizational strategy development) and has evidence for its feasibility and acceptability among mental health team members (Aarons et al., 2015). Financial strategies, tailored to the funding context for each setting, that facilitate sustained investment in service delivery framework implementation also appear critical. For example, whereas time-limited grant funds may support initial adoption of PBIS, state-level PBIS implementers recommended identifying reliable (i.e., recurring) and diverse funding streams to support scale-up and sustainability (Gage et al., 2014). Federal Title IV, Part A funds from the Every Student Succeeds Act of 2015 may be used for this purpose (von Ravensberg, 2020). However, prior research on complex innovations in primary care settings suggests that strategies targeting individual professionals are more often used and evaluated than those targeting organizations or context (Lau et al., 2015). Thus, it is imperative that implementation researchers continue to broaden their focus to specify (Proctor et al., 2013) and test implementation strategies that target aspects of the inner and outer organizational contexts.

### Limitations and Future Directions

Despite the potential of this conceptual paper to inform multilevel service delivery framework implementation, we acknowledge its limitations. Principally, we focused on three exemplar multilevel service delivery frameworks and did not complete a systematic review of the literature to identify core components and implementation determinants across the three exemplar frameworks included in this study. Our intention was to outline a conceptual foundation of commonalities and variation among framework components and implementation considerations that can span child mental health service settings. However, our focus on three exemplar frameworks (i.e., CoCM, PBIS, SoC), including one from each of three prominent youth mental health service settings, may have influenced the core components we identified as well as related implementation determinants. For example, it is possible that we may have identified additional or different core components had we focused on alternative frameworks (e.g., Interconnected Systems Framework) and/or service settings (e.g., juvenile justice). Similarly, relevant implementation determinants may also vary between frameworks and settings, such that frameworks that are emerging or less widely adopted may present with unique implementation challenges. Thus, our findings may not generalize to other multilevel frameworks or other settings. Research that expands upon this study to include additional frameworks, including those that are less widespread in their adoption and implementation, is needed. In addition, the foundation we articulated here will benefit from further refinement and elaboration through systematic reviews and coding of the bodies of literature for the exemplar multilevel service delivery frameworks; the components identified in this study should be considered preliminary until that work is completed. Reviews that articulate core components of each service delivery framework and the factors affecting their implementation (see Wood et al., 2017 for an example), and that are able to bridge across service settings to improve interdisciplinary implementation, are needed.

We highlighted prominent implementation determinants for each multilevel framework core component and coded these determinants using the CFIR. However, we did not engage in a systematic review process to identify these determinants. Determinants were identified from a range of articles and originated from study data or were extracted from manuscript text (e.g., results, discussion sections). Future systematic reviews of the literature that identify determinants from empirical evaluations of implementation barriers, facilitators, and outcomes are needed to more conclusively indicate which determinants are consequential in affecting implementation outcomes. Additionally, although the CFIR is effective for identifying and coding determinants across settings, we may have identified different determinants or augmented our discussion of relevant determinants had we used another framework (e.g., Theoretical Domains Framework; Cane et al., 2012). For example, the interplay between inner and outer setting factors that affects the cross-linking of service sectors as part of multilevel service delivery framework implementation can be represented by bridging factors in the Exploration, Preparation, Implementation, and Sustainment (EPIS) framework (Lengnick-Hall et al., 2021). Implementation of this component of multilevel frameworks may be further supported by additional research that investigates potential bridging factors.

Finally, identifying implementation determinants related to the core components of each framework was also challenging due to variability in terminology and focus in the relevant bodies of literature. Despite an acknowledged implementation gap across child mental health service settings, the application of implementation science and its associated terminology has progressed at variable rates across settings. For example, multiple scholars have acknowledged the underutilization of implementation science to date in schools compared to other settings (Forman et al., 2013; Sanetti & Collier-Meek, 2019). Though discussed in relation to overall framework implementation, implementation determinants were rarely discussed in alignment with core features of PBIS or SoC nor connected to implementation frameworks. This discrepancy highlights the importance of efforts to increase implementation science literacy outside of traditional health care settings (e.g., Sanetti & Collier-Meek, 2019), as well as the urgent opportunity to leverage interdisciplinary knowledge and collaboration to advance multilevel service delivery framework implementation. Researchers of PBIS, for example, might draw from the CoCM literature to inform and advance their own work related to PBIS implementation.

## Conclusion

The goals of this paper were to integrate the diverse literatures on widely used multilevel service delivery frameworks in child mental health services as well as to serve as a starting point for cross-sector and interdisciplinary scholarship and practice by specifying common core components and multilevel implementation determinants that interact with each component. Our hope is that this foundation can inform efforts to design and evaluate implementation strategies capable of addressing the multitude of barriers to multilevel service delivery framework implementation and which are tailored to these frameworks’ core features and context. Improving the high-quality implementation of multilevel service delivery frameworks may ultimately support increased access to high-quality mental health care for youth across a wide range of settings.

## Footnotes

1 We use the general terms “provider” or “professional” to refer to individuals who are involved in the implementation of multilevel service delivery frameworks. However, we recognize that terminology used to describe partners involved in implementation may vary across frameworks, for example, including peer supporters and other implementers.
